# Objective Auscultation of TCM Based on Wavelet Packet Fractal Dimension and Support Vector Machine

**DOI:** 10.1155/2014/502348

**Published:** 2014-05-05

**Authors:** Jian-Jun Yan, Rui Guo, Yi-Qin Wang, Guo-Ping Liu, Hai-Xia Yan, Chun-Ming Xia, Xiaojing Shen

**Affiliations:** ^1^Center for Mechatronics Engineering, East China University of Science and Technology, Shanghai 200237, China; ^2^Laboratory of Information Access and Synthesis of TCM Four Diagnostic, Shanghai University of Chinese Traditional Medicine, Shanghai 201203, China

## Abstract

This study was conducted to illustrate that auscultation features based on the fractal dimension combined with wavelet packet transform (WPT) were conducive to the identification the pattern of syndromes of Traditional Chinese Medicine (TCM). The WPT and the fractal dimension were employed to extract features of auscultation signals of 137 patients with lung Qi-deficient pattern, 49 patients with lung Yin-deficient pattern, and 43 healthy subjects. With these features, the classification model was constructed based on multiclass support vector machine (SVM). When all auscultation signals were trained by SVM to decide the patterns of TCM syndromes, the overall recognition rate of model was 79.49%; when male and female auscultation signals were trained, respectively, to decide the patterns, the overall recognition rate of model reached 86.05%. The results showed that the methods proposed in this paper were effective to analyze auscultation signals, and the performance of model can be greatly improved when the distinction of gender was considered.

## 1. Introduction

As one of the four diagnostic methods of Traditional Chinese Medicine (TCM), auscultation identifies a syndrome or disease by listening to the speech of patients. Auscultation method was definitely proposed in the Internal Classic of Huang Di [1]. However, it was not until after the Ming and Qing Dynasties that this method attracted attention from the medical field and both its theoretical and clinical application underwent much development. Since then, auscultation has become a distinctive step-by-step diagnostic method. In TCM, auscultation mainly depends on the auditory senses of the physician to accurately identify asthenia, sthenia, and visceral lesions in the patient. Therefore, auscultation is considered a qualitative method that produces unconvincing results.

Objective studies on auscultation have made some progress with the recent developments in computer and signal processing technologies [2]. Frequency spectrum analysis was made on the voice of cough patients making use of digital sonograph [3], but it cannot be quantitatively analyzed and applied for clinical diagnosis. The survey about objective auscultation of TCM was presented in [4], in which the digital technology with respect to auscultation was described and analyzed. The nonlinearity of the vowel /a:/ signals of healthy persons and patients with deficiency syndrome was investigated by using delay vector variance [5], whose studies were an effective attempt on the objective auscultation research. Chiu et al. proposed four novel acoustic parameters, the average number of zero-crossings, the variations in local peaks and valleys, the variations in first and second formant frequencies, and the spectral energy ratio to analyze and identify the characteristics among nondeficiency, Qi-deficiency, and Yin-deficiency subjects [6]. The energy values of wavelet packet coefficients were extracted for the auscultation signals of healthy people and patients with five Zang-organs diseases, and the results were analyzed and discussed [7, 8]. Wavelet packet transform (WPT) and Sample Entropy were combined together to analyze the auscultation signals in TCM. Sample Entropy values for WPT coefficients reflecting the complexity of the signal in different time-frequency resolutions were computed to quantify the signals from three kinds of samples including Qi-deficiency, Yin-deficiency, and healthy people. The statistical and classification results indicated that the method is useful and effective in auscultation research [9].

These studies provide a good basis for the objective research on its clinical use. However, these studies are still in their initial stages, the experiments are usually carried out in limited conditions, the adopted auscultation signals are not typical and comprehensive enough, and the algorithms are unreliably conducted on a small sample database. Therefore, the creation of a reliable and accurate algorithm remains a challenge in auscultation research.

Speech production is a complex process to analyze through traditional methods as it is influenced by glottis, track, and radiation [10]. The nonlinear characteristics of speech, such as the changes in the voice of the speaker when pronouncing the same phoneme twice, also hinder its analysis. Moreover, the predicted signal cannot accurately match the original speech regardless of the linear production order. Pronunciation includes both the nonlinear libration process and the speech itself. Changes in the shape of the tongue and track, particularly in spirant and explodent speeches, can produce a whirlpool on the track boundary layer that subsequently becomes a chaotic onflow [11]. The time-domain waveform of speech is self-comparable, and it also observes the characteristics of periodicity and randomicity. These findings show that chaos and fractal theories can be used in speech signal analysis [12].

Speech analysis must also be improved. The chaotic and fractal characteristics of speech suggest the combination of the nonlinear fractal dimension and the wavelet packet transform (WPT) to solve the problems in speech signal analysis. This study employed fractal dimension with WPT to analyze the auscultation signals of patients with chronic bronchitis as research object. The patients with chronic bronchitis occurred mostly in the pattern of lung Qi-deficiency and the pattern of lung Yin-deficiency. Therefore, this study analyzed the auscultation signals of patients with lung Qi-deficient pattern, patients with Yin-deficient pattern, and healthy subjects as control. Statistical analysis was made to obtain the effective features, which were used in the multiclass support vector machine (SVM) classifiers. Classification models were constructed to automatically identify the auscultation samples. The classification results are discussed at the end of the study.

## 2. Materials and Methods

### 2.1. Collected Materials

Patients who met the diagnostic criteria of chronic bronchitis and provided informed consent were included in the present study. The diagnostic criteria of patients were based on western medicine and TCM. The diagnostic criteria based on western medicine were adopted “the chronic bronchitis clinical diagnosis and curative effect judgment standard” [13]. The diagnostic criteria based on TCM were according to “Guideline for Clinical Study on New Drugs of Traditional Chinese Medicine” [14] and “Clinic Terminology of Traditional Chinese Medical Diagnosis and Treatment-Syndromes” [15], which is the national standard made by the State Bureau of Technical Quality Supervision, as well as the standards in textbooks [16, 17]. Patients with other severe disease, as well as those who cannot express their feelings clearly and did not provide informed consent, were excluded in the present study.

A total of 229 subjects were collected by TCM Syndrome Lab in Shanghai University of Traditional Chinese Medicine. The 186 patients with chronic bronchitis from the affiliated hospital of Shanghai University of TCM were separated into two groups, namely, lung Qi-deficient subjects (Group Q) and lung Yin-deficient subjects (Group Y). Fourty-three healthy subjects from the faculty of the Shanghai University of TCM were in control group (Group H). The detailed information is listed in Table 1. Lung Qi-deficiency and lung Yin-deficiency are TCM specific terms. The pattern of lung Qi-deficiency refers to the condition of declining function of the lung in governing Qi and defending the exterior. Its clinical manifestations are weak cough, panting, spitting of clear and thin phlegm, laziness in speaking, fatigue and a pale complexion, and other signs and symptoms. The pattern of lung Yin-deficiency refers to lung-yin failing to disperse and descend and an internal production of deficient-heat. Its clinical manifestations are dry cough or cough with scanty and sticky sputum or even with blood-streaked sputum, a dry mouth and throat, emaciation, a feverish sensation in the palms, night sweats, red cheeks and a hoarse voice, and other signs and symptoms.

The speech signals of the three groups were recorded using a microphone and were digitized using a 24-bit A/D acquisitive card (Brand CME Xcorpio, with a frequency response range of 20 Hz to 20 kHz and a dynamic range of 100 dB) at a 16 KHz sampling rate with an antialiasing function. The speech signals were recorded at a maintained collecting distance and position. The vowel /a:/ is easy for either patients or healthy people to pronounce. In addition, the vocal organ is not abuttal and there is no obstacle in cavity when someone is sending out the vowel [18]. Thus, each patient was asked to utter the vowel /a:/. Each subject produced a sustained stable phonation of vowel /a:/ that lasted for about 1 second.

The research was conducted at Shanghai University of TCM and its affiliated hospital after the approval of the moral and ethical committee of Shanghai University of TCM and informed consent of all subjects had been obtained.

### 2.2. Methods

#### 2.2.1. Wavelet Packet Decomposition Algorithm

Wavelet transform is a time-frequency analysis method that uses different scales to obtain the best time-domain and frequency-domain resolutions in different parts of the signal. Resolution analysis conducts further decomposition in the low frequency part only to prevent the subdivision of the high frequency part [19, 20]. WPT provides a more precise decomposition for the signal analysis and carries out further decomposition in the high frequency part to subdivide the frequency bands in the low and high frequency parts synchronously. WPT can also self-adaptively select the signal resolution in different frequency bands to improve the time-frequency resolution [21–23].

**Figure 1:**
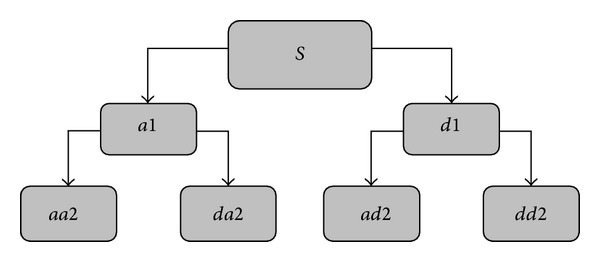
Tree of two-layer WPD.

**Figure 2:**
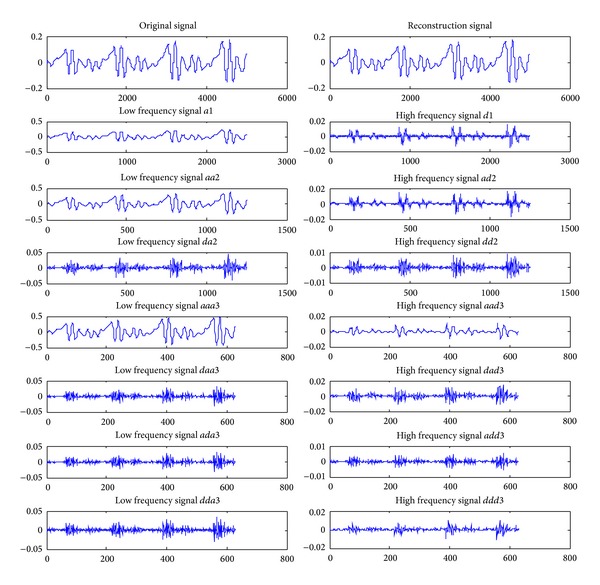
Decomposed and reconstructed waveform of a certain voice signal.

A common wavelet function consists of the Harr wavelet, Daubechies wavelet, SymletsA wavelet, Coiflet wavelet, Morlet wavelet, and Mexican Hat wavelet. After comparing the analysis results of db, coif, and sym wavelet functions, the db4 wavelet function with a high energy concentration was ultimately chosen for the analysis of the speech signals of the three sample groups [24].  Figure 1 shows the two-layer WPD tree, and Figure 2 shows the decomposition of a speech signal at layer 3, where  *S*  is the original signal,  *a*  represents the approximations (the low frequency components), and  *d*  represents the details (the high frequency components).

#### 2.2.2. Fractal Dimension

Although the fractal dimension has several definitions [25], the box dimension definition is used in this paper for calculation convenience. Time sequence  *A*  of the speech signal is covered with a reticulation grid.  *S*  represents the border length, and  *N*(*S*)  denotes the number of mesh grids that contain set  *A*  [26]. The box dimension definition is represented by the following equation:

(1) $$\begin{array}{c} D_{B}=\underset{s\to 0}{\sum }\frac{\log N\left(S\right)}{\log\left(1/S\right)}. \end{array}$$

The least square method (LSM) is used to approximate the log⁡*N*(*s*) ~ log⁡(1/*s*)  line. The slope is represented by box dimension  *D*_*B*_. The approximation is carried out as follows.

(1) The original speech is unified to a unit square area with a gain signal of  *x*(*t*),  *x*(*t*) ≤ 1.
(2) The square area is divided into the mesh grids, and the log⁡*N*(*s*)  and  log⁡(1/*s*)  are calculated. The change in  *S*  is recorded, and the corresponding log⁡*N*(*s*)  and  log⁡(1/*s*)  are calculated.
(3) Let  *x*_*i*_ = log⁡(1/*s*_*i*_) and *y*_*i*_ = log⁡*N*(*s*_*i*_),   *i* = 1, 2,…, *M*.   (*x*_*i*_, *y*_*i*_)  is used to approximate line  *y* = *kx* + *b*  by LSM.  *k*  represents the box dimension  *D*_*B*_, which is calculated as follows:
  (2) $$\begin{array}{c} D_{B}=\frac{\left(\sum_{i=1}^{M}y_{i}\right)\left(\sum_{i=1}^{M}x_{i}\right)-M\left(\sum_{i=1}^{M}y_{i}x_{i}\right)}{{\left(\sum_{i=1}^{M}x_{i}\right)}^{2}-M\left(\sum_{i=1}^{M}{x_{i}}^{2}\right)}. \end{array}$$

#### 2.2.3. Wavelet Packet Fractal Dimension

Fractal theory is found to be in agreement with the wavelet analysis in terms of self-similarity and understanding things from coarse to fine scales [27]. This paper proposes a wavelet packet fractal theory that uses the WPD of the auscultation signals to compare the box dimension value and its changes in different frequency bands to reflect the irregularity, complexity, and nonstationarity of the signals. The box dimension values of the discrete signals range from 1 to 2, indicating that the more complex the signals are, the greater the box dimension values become.

#### 2.2.4. Support Vector Machine

SVMs were first introduced by Vapnik (1998) to perform highly effective classification, regression, and pattern recognition processes [28]. SVM uses a hypothesis space of linear functions in a high-dimensional space and is trained with a learning algorithm from optimization theory that implements a learning bias from statistical learning theory. SVM uses a linear model to implement nonlinear class boundaries by nonlinearly mapping input vectors into a high-dimensional feature space using kernels [29, 30]. Previous studies suggest using the Radial Basis Function (RBF) kernel as a default kernel. The kernel parameters can be automatically chosen by optimizing a cross-validation-based model selection. One-against-one (1-versus-1) and one-against-rest (1-versus-r) are two popular multi-SVM schemes. In a  *k*-class problem, 1-versus-1 forms a training subset for every possible class pair combination and learns an SVM model from each subset. A total of  *k* × (*k* − 1)/2  SVM classifiers are trained for all the combinations. The class for an unseen example is obtained by majority vote of all binary SVM classifiers. The classification result is affected if the same kernel parameters are used in all the SVM classifiers that apply the 1-versus-1 scheme. Therefore, the kernel parameters of each SVM classifier must be separately identified through cross-validation. A multiclass SVM classifier is used to identify the auscultation signals. Each SVM classifier chooses specific RBF kernel parameters through a grid search with a nested cross-validation [31].

## 3. Experimental Results

### 3.1. Feature Analysis

The auscultation signals were processed by a self-developed analytic program under the Matlab environment. The decomposition structure tree at layer 7 was used for the WPD of the auscultation signals in the computer experiment.  2^*i*^  frequency subbands were observed at the  *i*th layer after the WPD of the speech signals. The fractal dimensions of the speech voice in each subband were calculated with a sampling rate of 16,000 Hz and 16 bit precision. The frequency bands for these subbands are as follows: first layer (frequency interval = 4 kHz,  *n*  = 0, 1), second layer (frequency interval = 2 kHz,  *n*  = 0, 1, 2, 3), third layer (frequency interval = 1 kHz,  *n* = 0, 1, 2,…, 7), fourth layer (frequency interval = 0.5 kHz,  *n* = 0, 1, 2,…, 15), and fifth layer (frequency interval = 0.25 kHz,  *n* = 0, 1, 2,…, 31). After calculating the box dimensions of vowel /a:/ and observing the fractal dimension trajectory curve, the following findings were made.

**Figure 3:**
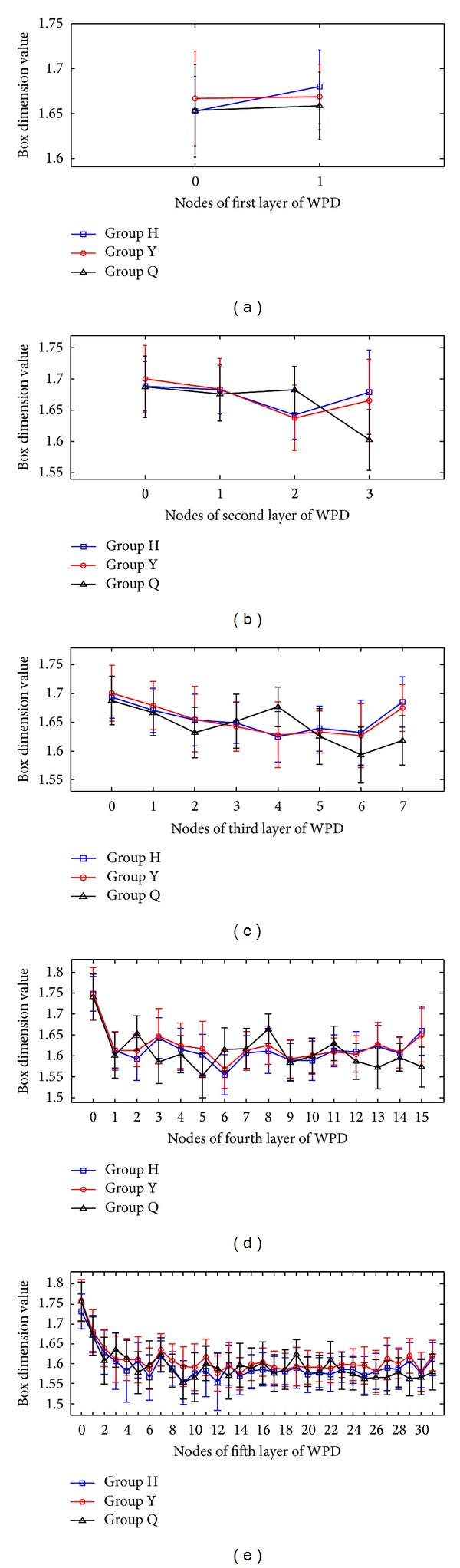
Box dimension values of the coefficients of WPT decomposition for all subjects: (a–e) box dimension values for the first to the fifth layer coefficients.

**Figure 4:**
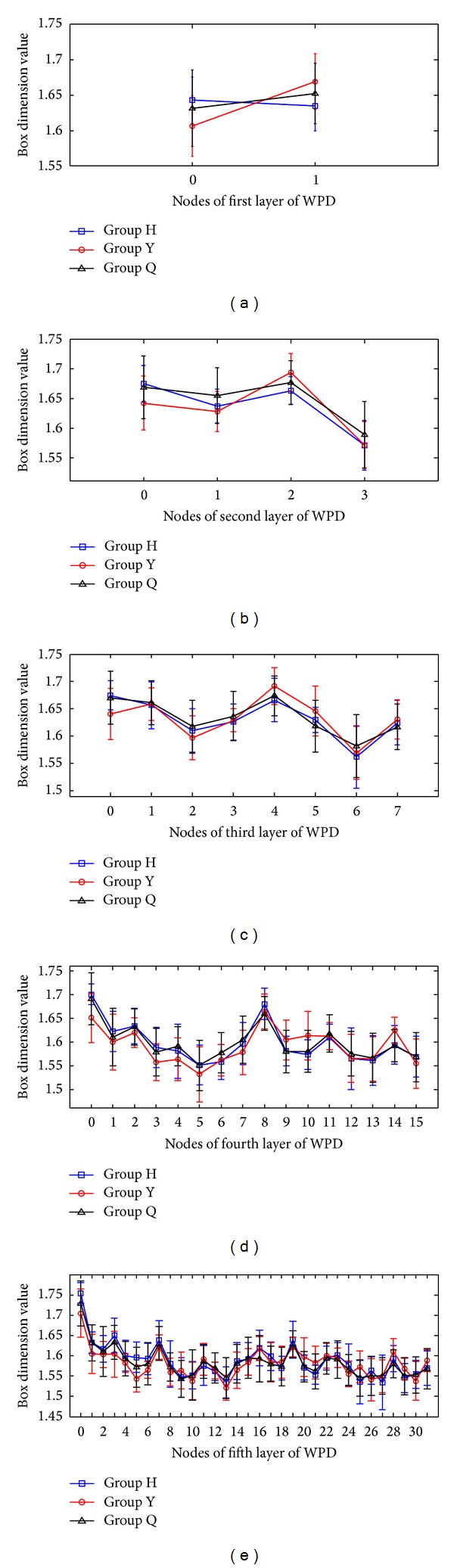
Box dimension values of the coefficients of WPT decomposition for male subjects: (a–e) box dimension values for the first to the fifth layer coefficients.

**Figure 5:**
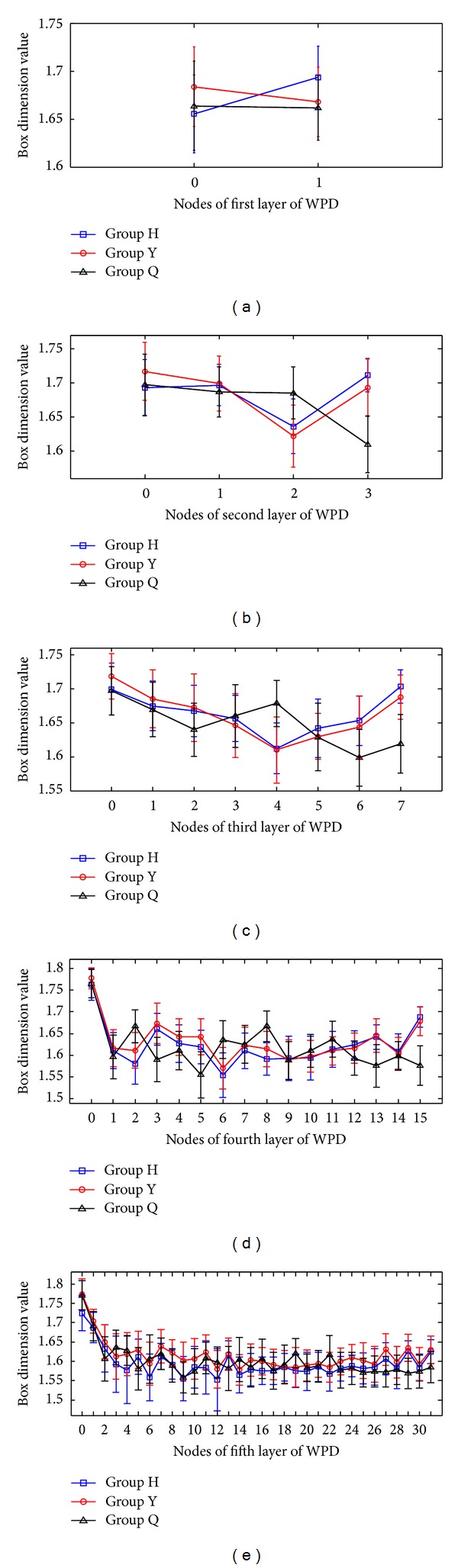
Box dimension values of the coefficients of WPT decomposition for female subjects: (a–e) box dimension values for the first to the fifth layer coefficients.

1. The box dimension value in each subband ranges from 1.3 to 1.8, indicating the existence of a regular fractal dimension space distribution for the auscultation signals. This result also indicates that the box dimension value of auscultation signals is completely different from that of noise.
2. The auscultation signals of male subjects are different from those of female subjects.
3. Different auscultation signals have different box dimension values, and the box dimension values between the first layer and the second layer of WPD significantly differ. Figures 3 to 5 show different trends in the box dimension values between the first layer and the fifth layer subbands.

### 3.2. Statistical Analysis

The statistical analysis software SPSS 20 was used to analyze the differences between the samples. Age as covariant was included in the statistic model to correct the effect of age. The box dimension values of the WPT coefficients from the first to the fifth layers were analyzed to identify the significant differences between the two groups of samples.  Table 2 shows 43 frequency bands with significantly different box dimension values from the first to fifth layers in all subjects.  Table 3 shows 7 frequency bands with significantly different box dimension values from the first to fifth layers in the male subjects.  Table 4 shows 48 frequency bands with significantly different box dimension values from the first to fifth layers in the female subjects.

### 3.3. Classification

The multiclass SVM was applied as a classifier to discriminate Group Q, Group Y, and Group H by the extracted features of box dimension. The RBF function was chosen as the kernel function. The classification model, in which every binary classifier had its own feature subset and RBF kernel parameters, was constructed to identify the auscultation signals based on the libsvm software [32].

In the process of classification, threefold cross-validation was applied on the classification of auscultation signals. For comparison of recognition accuracy, the experiments were done with different training data. The classification results using multiclass SVM were shown in Table 5. When the auscultation signals of all subjects were trained by SVM to decide the patterns of TCM syndromes, the overall accuracy of all subjects was 79.49%. When auscultation signals of male and female auscultation signals were trained, respectively, to decide the patterns of TCM syndromes, the overall accuracy of male subjects was 91.95% and that of female subjects was 72.07%. And the overall accuracy of male and female subjects was 86.05%, which was the weighted accuracy and the weight was the proportion of subject number of the group with different gender in all samples.

## 4. Discussions

In TCM, the signs and symptoms can be captured through four methods of diagnosis, namely, inspection, auscultation and olfaction, inquisition, and pulse taking. Combined use of four methods is necessary for acquiring full and detailed clinical information as well as disease diagnosis. Auscultation is indispensable part of four methods. The traditional auscultation depends on subjective hearing; however, accurate auscultation is difficult to be done by TCM doctors who lack experience and have decrease in hearing acuity. Therefore, objective research of auscultation is highly desirable, which contributes to quantitatively combined use of four methods, avoiding clinical misdiagnosis or missed diagnosis.

Box dimension  *D*_*B*_  can be used for analysis and classification in auscultation research. Independent from  *D*_*B*_, the auscultation signal shows a relatively steady fractal dimension value, and the dimension values among the subbands show significant differences. Figures 3(b)–3(d) and Table 2 show that Group H and Group Y share the same variations in their box dimension values. The box dimension values of Group H and Group Y for all subjects are the largest in the low frequency band, then those become lower, and finally the box dimension values of all groups rise to certain extent in the high frequency band. However, the box dimension value of Group Q of all subjects is higher, and then it fluctuates in the other frequency bands. As shown in Figures 4(b)–4(d) and Table 3, Group H has the similar variety of box dimension values to Group Q instead of Group Y. The box dimension values of all groups for all subjects are higher in the low frequency band. In addition, there is a peak in the frequency band around 4 kHz. However, the change of box dimension values of Group H and Group Y for female subjects in Figure 5 is similar to that in Figure 3. As shown in Tables 2 to 4, most frequency bands with significantly different box dimension values in female subjects, while least frequency bands with significantly different box dimension values in male subjects. It indicates that the female subjects have better class separability in wavelet packet fractal dimensions than the male subjects and all subjects.

The internal information on the auscultation signals shows some irregularities, and the fractal dimension trajectory distinctively varies among the subjects. The differences in the trends are reflected in the diverse internal information of the three groups, and this information can be applied for clinical diagnosis in TCM.

As shown in Table 5, when auscultation signals of all subjects were trained by classification model to decide the patterns of TCM syndromes, the overall accuracy was 79.49%. When those of male subjects were trained by classification model the accuracy was 72.07%, whereas the overall accuracy was up to 91.95% when those of female subjects were trained. Therefore, the overall accuracy of male and female subjects reached 86.05%. The resulted showed that the methods proposed in this paper were effective to analyze auscultation signals, and the performance of model can be greatly improved when distinction of gender was considered.

## 5. Conclusions

This paper selected the vowel /a:/ to be pronounced by each subject to decrease the interference, complexity, and uncertainty of the auscultation signal analysis. The auscultation signals were further processed using combination of box dimension and wavelet packet transform by self-developed analytic program under Matlab environment. Then the box dimension values of the auscultation signals were analyzed and compared in each frequency subband. The differences in the trends are reflected in the diverse internal information of the three groups, and this information was applied for the further classification of auscultation signals. The male and female classification models, if established separately, are applicable and effective in the auscultation analysis of TCM. The clinical subject size must be extended for future studies to verify our proposed methods. Our future research aims to construct an automatic auscultation system to assist in clinical diagnosis.

## Figures and Tables

**Table 1:** The group, sex, and age of all subjects in the experiments.

	Group H	Group Y	Group Q
Subjects			
Male	10	11	47
Female	33	38	90
Total	**43**	**49**	**137**
Age (mean ± std)	26.3 ± 6.2	53.5 ± 10.6	45.2 ± 12.5

**Table 2:** Significant difference between the box dimension values for the coefficients of the subbands from the first to fifth layers in all subjects.

Node	Frequency band (KHz)	Group H (mean ± std)	Group Y (mean ± std)	Group Q (mean ± std)
1.1	4–8	1.680 ± 0.041^#^	1.668 ± 0.037	1.659 ± 0.037*
2.2	4–6	1.642 ± 0.038^#^	1.638 ± 0.053^#^	1.683 ± 0.038^∗∧^
2.3	6–8	1.679 ± 0.067^#^	1.666 ± 0.066^#^	1.603 ± 0.048^∗∧^
3.0	0-1	1.694 ± 0.037	1.701 ± 0.049^#^	1.688 ± 0.042^∧^
3.2	2-3	1.654 ± 0.045^#^	1.655 ± 0.057^#^	1.632 ± 0.044^∗∧^
3.4	4-5	1.625 ± 0.044^#^	1.628 ± 0.057^#^	1.677 ± 0.035^∗∧^
3.6	6-7	1.632 ± 0.057^#^	1.627 ± 0.055^#^	1.593 ± 0.048^∗∧^
3.7	7-8	1.685 ± 0.044^#^	1.675 ± 0.041^#^	1.618 ± 0.043^∗∧^
4.2	1–1.5	1.593 ± 0.051^#^	1.613 ± 0.039^#^	1.655 ± 0.041^∗∧^
4.3	1.5–2	1.643 ± 0.049^#^	1.647 ± 0.066^#^	1.586 ± 0.051^∗∧^
4.4	2–2.5	1.616 ± 0.050	1.624 ± 0.054^#^	1.604 ± 0.044^∧^
4.5	2.5–3	1.603 ± 0.048^#^	1.618 ± 0.065^#^	1.553 ± 0.053^∗∧^
4.6	3–3.5	1.555 ± 0.048^#^	1.568 ± 0.045^#^	1.616 ± 0.051^∗∧^
4.8	4–4.5	1.612 ± 0.053^#^	1.626 ± 0.045^#^	1.665 ± 0.035^∗∧^
4.10	5.5–6	1.613 ± 0.038^#^	1.611 ± 0.032^#^	1.630 ± 0.041^∗∧^
4.11	6–6.5	1.611 ± 0.048^#^	1.605 ± 0.044^#^	1.587 ± 0.043^∗∧^
4.12	6.5–7	1.623 ± 0.049^#^	1.627 ± 0.052^#^	1.573 ± 0.050^∗∧^
4.14	7.5–8	1.661 ± 0.058^#^	1.650 ± 0.064^#^	1.574 ± 0.048^∗∧^
5.0	0–0.25	1.731 ± 0.044^#^	1.758 ± 0.053	1.757 ± 0.049*
5.2	0.5–0.75	1.629 ± 0.056^#^	1.639 ± 0.046^#^	1.607 ± 0.059^∗∧^
5.3	0.75–1	1.606 ± 0.071^#^	1.611 ± 0.058^#^	1.635 ± 0.044^∗∧^
5.4	1–1.25	1.583 ± 0.079^#^	1.610 ± 0.053	1.616 ± 0.044*
5.5	1.25–1.5	1.608 ± 0.044^#^	1.610 ± 0.058^#^	1.578 ± 0.053^∗∧^
5.6	1.5–1.75	1.566 ± 0.058^#^	1.587 ± 0.052	1.597 ± 0.060*
5.7	1.75–2	1.619 ± 0.039^∧^	1.635 ± 0.041*	1.623 ± 0.042
5.8	2–2.25	1.588 ± 0.042^∧^	1.608 ± 0.042^∗#^	1.583 ± 0.042^∧^
5.9	2.25–2.5	1.553 ± 0.055^∧^	1.593 ± 0.050^∗#^	1.553 ± 0.043^∧^
5.10	2.5–2.75	1.577 ± 0.052	1.591 ± 0.059^#^	1.566 ± 0.061^∧^
5.11	2.75–3	1.582 ± 0.064^∧^	1.616 ± 0.046^∗#^	1.601 ± 0.043^∧^
5.12	3–3.25	1.554 ± 0.071^#^	1.576 ± 0.044^#^	1.588 ± 0.042^∗∧^
5.13	3.25–3.5	1.597 ± 0.051^#^	1.597 ± 0.057^#^	1.569 ± 0.057^∗∧^
5.14	3.5–3.75	1.569 ± 0.047^#^	1.575 ± 0.041^#^	1.597 ± 0.055^∗∧^
5.16	4–4.25	1.586 ± 0.042^∧#^	1.604 ± 0.035*	1.602 ± 0.053*
5.19	4.75–5	1.590 ± 0.051^#^	1.593 ± 0.048^#^	1.624 ± 0.036^∗∧^
5.21	5.25–5.5	1.578 ± 0.038	1.591 ± 0.036^#^	1.578 ± 0.044^∧^
5.22	5.5–5.75	1.573 ± 0.043^#^	1.589 ± 0.037^#^	1.609 ± 0.047^∗∧^
5.23	5.75–6	1.586 ± 0.032^∧^	1.599 ± 0.032^∗#^	1.582 ± 0.046^∧^
5.24	6–6.25	1.585 ± 0.035	1.598 ± 0.040^#^	1.575 ± 0.041^∧^
5.25	6.25–6.5	1.570 ± 0.047^∧^	1.595 ± 0.046^∗#^	1.562 ± 0.041^∧^
5.27	6.75–7	1.590 ± 0.057^#^	1.612 ± 0.053^#^	1.565 ± 0.043^∗∧^
5.28	7–7.25	1.586 ± 0.051	1.601 ± 0.039^#^	1.579 ± 0.037^∧^
5.29	7.25–7.5	1.608 ± 0.045^#^	1.620 ± 0.044^#^	1.562 ± 0.043^∗∧^
5.31	7.75–8	1.612 ± 0.041^#^	1.620 ± 0.038^#^	1.579 ± 0.045^∗∧^

Compared with Group H, *P < 0.05. Compared with Group Y, ^∧^ *P* < 0.05. Compared with Group Q, ^#^ *P* < 0.05.

**Table 3:** Significant difference between the box dimension values for the coefficients of the subbands from the first to fifth layers in male subjects.

Node	Frequency band (KHz)	Group H (mean ± std)	Group Y (mean ± std)	Group Q (mean ± std)
2.2	4–6	1.663 ± 0.024^∧^	1.694 ± 0.032*	1.677 ± 0.037
4.0	0–0.5	1.701 ± 0.022^∧^	1.651 ± 0.052^∗#^	1.691 ± 0.055^∧^
4.4	2–2.5	1.581 ± 0.057	1.564 ± 0.045^#^	1.592 ± 0.042^∧^
4.10	5–5.5	1.573 ± 0.031	1.614 ± 0.052^#^	1.580 ± 0.045^∧^
4.14	7–7.5	1.594 ± 0.041	1.625 ± 0.028^#^	1.592 ± 0.034^∧^
5.1	0.25–0.5	1.755 ± 0.026^∧^	1.704 ± 0.059*	1.729 ± 0.056
5.5	1.25–1.5	1.596 ± 0.037^∧^	1.544 ± 0.033^∗#^	1.572 ± 0.049^∧^

Compared with Group H, *P < 0.05. Compared with Group Y, ^∧^ *P* < 0.05. Compared with Group Q, ^#^ *P* < 0.05.

**Table 4:** Significant difference between the box dimension values for the coefficients of the subbands from the first to fifth layers in female subjects.

Node	Frequency band (KHz)	Group H (mean ± std)	Group Y (mean ± std)	Group Q (mean ± std)
1.0	0–4	1.656 ± 0.041^∧^	1.684 ± 0.042^∗#^	1.664 ± 0.047^∧^
1.1	4–8	1.694 ± 0.033^∧#^	1.668 ± 0.036*	1.662 ± 0.034*
2.0	0–2	1.693 ± 0.041^∧^	1.717 ± 0.043^∗#^	1.698 ± 0.045^∧^
2.2	4–6	1.636 ± 0.040^#^	1.622 ± 0.046^#^	1.686 ± 0.038^∗∧^
2.3	6–8	1.712 ± 0.025^∧#^	1.693 ± 0.042^∗#^	1.610 ± 0.042^∗∧^
3.0	0-1	1.699 ± 0.038	1.718 ± 0.033^#^	1.697 ± 0.036^∧^
3.2	2-3	1.668 ± 0.038^#^	1.672 ± 0.050^#^	1.640 ± 0.039^∗∧^
3.4	4-5	1.612 ± 0.037^#^	1.610 ± 0.049^#^	1.678 ± 0.034^∗∧^
3.6	6-7	1.653 ± 0.036^#^	1.644 ± 0.045^#^	1.599 ± 0.042^∗∧^
3.7	7-8	1.704 ± 0.025^∧#^	1.688 ± 0.033^∗#^	1.619 ± 0.043^∗∧^
4.2	1–1.5	1.581 ± 0.048^∧#^	1.611 ± 0.041^∗#^	1.667 ± 0.038^∗∧^
4.3	1.5–2	1.66 ± 0.037^#^	1.673 ± 0.046^#^	1.590 ± 0.051^∗∧^
4.4	2–2.5	1.627 ± 0.043^#^	1.642 ± 0.042^#^	1.611 ± 0.044^∗∧^
4.5	2.5–3	1.619 ± 0.038^∧#^	1.642 ± 0.042^∗#^	1.555 ± 0.053^∗∧^
4.6	3–3.5	1.554 ± 0.051^#^	1.570 ± 0.048^#^	1.636 ± 0.043^∗∧^
4.8	4–4.5	1.591 ± 0.038^∧#^	1.615 ± 0.041^∗#^	1.667 ± 0.035^∗∧^
4.10	5–5.5	1.594 ± 0.050^#^	1.598 ± 0.036	1.611 ± 0.038*
4.11	5.5–6	1.613 ± 0.041^#^	1.610 ± 0.033^#^	1.637 ± 0.041^∗∧^
4.12	6–6.5	1.624 ± 0.032^#^	1.616 ± 0.034^#^	1.593 ± 0.040^∗∧^
4.13	6.5–7	1.642 ± 0.029^#^	1.645 ± 0.038^#^	1.576 ± 0.049^∗∧^
4.15	7.5–8	1.688 ± 0.024^#^	1.678 ± 0.033^#^	1.577 ± 0.046^∗∧^
5.0	0–0.25	1.724 ± 0.046^∧#^	1.773 ± 0.040*	1.771 ± 0.038*
5.1	0.25–0.5	1.687 ± 0.040^∧^	1.703 ± 0.031^∗#^	1.691 ± 0.038^∧^
5.2	0.5–0.75	1.633 ± 0.061^#^	1.650 ± 0.044^#^	1.606 ± 0.058^∗∧^
5.3	0.75–1	1.593 ± 0.072^#^	1.613 ± 0.059^#^	1.636 ± 0.045^∗∧^
5.4	1–1.25	1.577 ± 0.087^#^	1.619 ± 0.056	1.628 ± 0.039*
5.5	1.25–1.5	1.612 ± 0.046^#^	1.630 ± 0.048^#^	1.581 ± 0.055^∗∧^
5.6	1.5–1.75	1.558 ± 0.061^∧#^	1.594 ± 0.056*	1.606 ± 0.063*
5.7	1.75–2	1.612 ± 0.034^∧^	1.638 ± 0.043^∗#^	1.619 ± 0.041^∧^
5.8	2–2.25	1.591 ± 0.037^∧^	1.622 ± 0.033^∗#^	1.589 ± 0.043^∧^
5.9	2.25–2.5	1.555 ± 0.057^∧^	1.601 ± 0.049^∗#^	1.559 ± 0.040^∧^
5.10	2.5–2.75	1.584 ± 0.055	1.607 ± 0.053^#^	1.573 ± 0.059^∧^
5.11	2.75–3	1.584 ± 0.069^∧^	1.623 ± 0.045*	1.609 ± 0.041
5.12	3–3.25	1.552 ± 0.081^#^	1.580 ± 0.049^#^	1.597 ± 0.041^∗∧^
5.13	3.25–3.5	1.616 ± 0.037^#^	1.618 ± 0.042^#^	1.582 ± 0.057^∗∧^
5.14	3.5–3.75	1.564 ± 0.047^#^	1.578 ± 0.039^#^	1.605 ± 0.057^∗∧^
5.15	3.75–4	1.579 ± 0.042^∧^	1.603 ± 0.042^∗#^	1.585 ± 0.051^∧^
5.16	4–4.25	1.575 ± 0.036^∧#^	1.600 ± 0.036*	1.606 ± 0.051*
5.19	4.75–5	1.575 ± 0.043^#^	1.585 ± 0.051^#^	1.621 ± 0.038^∗∧^
5.22	5.5–5.75	1.567 ± 0.044^#^	1.585 ± 0.039^#^	1.617 ± 0.050^∗∧^
5.23	5.75–6	1.581 ± 0.031^∧^	1.600 ± 0.035^∗#^	1.577 ± 0.046^∧^
5.24	6–6.25	1.588 ± 0.029^∧^	1.610 ± 0.034^∗#^	1.581 ± 0.042^∧^
5.25	6.25–6.5	1.581 ± 0.040	1.602 ± 0.047^#^	1.571 ± 0.037^∧^
5.27	6.75–7	1.606 ± 0.042^∧#^	1.631 ± 0.041^∗#^	1.573 ± 0.040^∗∧^
5.28	7–7.25	1.584 ± 0.055	1.598 ± 0.041^#^	1.578 ± 0.038^∧^
5.29	7.25–7.5	1.626 ± 0.026^#^	1.635 ± 0.035^#^	1.569 ± 0.040^∗∧^
5.30	7.5–7.75	1.584 ± 0.035	1.593 ± 0.042^#^	1.573 ± 0.042^∧^
5.31	7.75–8	1.625 ± 0.031^#^	1.630 ± 0.036^#^	1.585 ± 0.041^∗∧^

Compared with Group H, *P < 0.05. Compared with Group Y, ^∧^ *P* < 0.05. Compared with Group Q, ^#^ *P* < 0.05.

**Table 5:** Model recognition accuracies of all subjects, male subjects, and female subjects (%).

Group	Male	Female	All
Group Q	93.47	100.00	97.81
Group Y	44.44	76.71	55.39
Group H	0.00	87.88	48.89
Total	**91.95**	**72.07**	**79.49**
